# Nerve growth factor inhibits TLR3-induced inflammatory cascades in human corneal epithelial cells

**DOI:** 10.1186/s12950-019-0232-0

**Published:** 2019-12-26

**Authors:** Huiyu Chen, Jing Zhang, Yiqin Dai, Jianjiang Xu

**Affiliations:** Department of Ophthalmology and Visual Science, Eye & ENT Hospital, NHC Key Laboratory of myopia (Fudan University); Shanghai Key Laboratory of Visual Impairment and Restoration, Shanghai Medical College of Fudan University, Shanghai, 200031 China

**Keywords:** Nerve growth factor, Toll-like receptor 3, Herpes simplex keratitis, Corneal inflammation, Oxidative injury

## Abstract

**Background:**

In herpes simplex epithelial keratitis, excessive TLR3-induced cellular responses after virus infection evoke inflammatory cascades that might be destructive to the host cornea. Nerve growth factor (NGF), a pluripotent neurotrophic factor with immune regulatory effect, was proved to be effective in Herpes simplex keratitis (HSK) treatment, although the detailed mechanisms remain unclear. This study aims to investigate the effects of NGF on modulating inflammatory responses triggered by TLR3 activation in human corneal epithelial cells (HCECs) in vitro.

**Methods:**

HCECs were stimulated with TLR3 agonist, poly(I:C), in the absence or presence of NGF. Cell viability and cytotoxicity were measured by a CCK-8 assay and LDH release assay, respectively. The activation of NF-κB signaling pathway was examined using immunofluorescence staining and western blotting. Levels of proinflammatory cytokines were determined by ELISA or RT-qPCR. ROS generation and 8-OHdG positive cells were examined by a fluorometric analysis.

**Results:**

It was shown that NGF significantly inhibited the generation of proinflammatory cytokines in HCECs triggered by TLR3 activation (*P* < 0.05), probably via suppressing NF-κB activation. NGF also impeded the upstream signal to initiate NF-κB activation by scavenging ROS by approximately 50% (*P* < 0.05). In addition, 8-OHdG positive cells were substantially attenuated by NGF treatment (*P* < 0.01).

**Conclusions:**

Taken together, this study indicates that NGF could inhibit TLR3-induced inflammatory cascades in HCECs, suggesting NGF as a potential therapeutic agent for HSK.

## Background

Herpes simplex keratitis (HSK), a disorder induced by herpes simplex virus 1 (HSV-1) infection of corneal epithelial and stromal layers, is a leading cause of human blindness in developed countries [1]. Over 60% of new HSK cases present as epithelial keratitis. Cornea epithelium is the first line that protects eyes from external stimuli. It recognizes pathogen-associated molecular patterns in response to virus infection via residential Toll-like receptors (TLRs) [2], and thereby triggers innate immune responses to protect cells against microbial pathogens [3]. Although the initiated inflammatory responses are essential to clear the virus from the infected tissue, excessive TLR-induced cellular responses also evoke inflammatory cascades, which might lead to overactive inflammation infiltrating in host cornea, resulting in scar formation and vision loss [4, 5].

HSV-1 was shown to elicit pro-inflammatory cytokines through sequential activation of TLR2, 3, and 9 in corneal epithelial cells [6]. Among them, TLR3 has been considered as the major mediator against virus infection [7, 8]. It recognizes double stranded RNA (dsRNA) that is generated during virus replication, or the synthetic mimic of dsRNA, named Polyinosinic-polycytidylic acid (poly (I:C)), eliciting the generation of Type I IFNs and proinflammatory factors through its downstream NF-κB pathway. Poly (I:C) is a well-recognized TLR3 agonist. Using TLR3-deficient cells and mice, *Lena* et al. successfully demonstrated that TLR3 conferred responsiveness and specificity to poly (I:C) [9]. Moreover, poly (I:C) could be directly bound by the extracellular domain of CD14, thereby facilitating poly (I:C) uptake and enhancing TLR3 activation [10]. Poly (I:C) triggered TLR3 activation, followed by downstream inflammatory cascades have been reported in many corneal pathologies [11–13]. Recently, emerging evidences showed that TLR3 could also trigger the generation of reactive oxygen species (ROS), an essential secondary messenger that was required for NF-κB activation, leading to innate immune response [14]. However, the role of ROS production during HSV-1 infection is complicated. A significant abundance of ROS could break redox homeostasis, which plays a role in chronic inflammation and tissue damage [15, 16]. Therefore, given the pivotal role of TLR3-induced ROS generation and downstream inflammatory responses in corneal epithelium injury post virus infection, therapeutics targeting this important step may benefit in efficiently attenuating HSK progression.

Nerve growth factor (NGF) is a well-known neurotrophic factor that promotes cell growth, differentiation, survival and cell death. It is also shown to present regulatory effects in several inflammatory scenarios, including multiple sclerosis, colitis, and UV irradiation [17]. Signals mediated by NGF are propagated by its two important receptors, the high-affinity receptor TrkA and the low-affinity non-selective transmembrane glycoprotein receptor p75^NTR^ [18]. Recently, an increasing body of evidences demonstrated that NGF also revealed immune-modulatory effects in corneal physiopathology. Topical administration of NGF was successively applied in corneal healing [19], neurotrophic keratitis [20], immune corneal ulcer [21], dry eye syndrome [22], corneal transplantation [23], and diabetic cornea [24], highlighting the therapeutic potential of NGF in treating corneal diseases with a strong immune component involved. In HSK, endogenous NGF was shown to inhibit HSK recurrence, supported by the evidence that systemic neutralization of endogenous NGF induced HSV-1 reactivation in a rabbit model of HSK [25]. Consistently, *Lambiase* et al. also demonstrated that topical administration of anti-NGF antibody exacerbated disease severity in HSK rabbits [26], whilst treatment with NGF promoted a remarkable amelioration that was comparable to the effect of acyclovir [19]. Clinical treatment with NGF eye drops was shown to induce complete healing in a HSK patient that was resistant to acyclovir treatment [20], further implicating the effectiveness of NGF in HSK treatment. However, the role of NGF in HSK has not been extensively studied yet, and the detailed mechanism underlying its cytoprotective effect on HSK induced cornea epithelium injury still remains unclear.

In this study, we investigated the inhibitory effect of NGF on TLR3 triggered cellular responses, a critical step associated with HSV-1 induced cornea injury. We sought to determine whether NGF could modulate inflammatory genes and related upstream pathways in TLR3 activated human corneal epithelial cells (HCECs).

## Materials and methods

### Human corneal epithelial cell (HCEC) culture

As previously described [27], the immortalized HCECs were cultured in the medium consisted of 90% DMEM/F12, 10% fetal bovine serum and 1% penicillin/streptomycin in 5% CO_2_ incubator at 37° for 24 h. Then HCECs were seeded in 6-, 24-, or 96-well plates at a density of 1*10^5^cells/mL adding different concentrations of poly (I:C) (Sigma p9582, USA) or NGF (Sigma SRP3015, USA) depending on the experiments. The whole research procedures were approved by the Ethics Committee of EENT Hospital of Fudan University and were performed following the declaration of Helsinki.

### Cell viability assay

A CCK-8 assay (Dojindo, Japan) was used to examine cell viability according to the manufacturer’s instructions. 10 μL of CCK-8 solution was added to each well of the plate and the absorbance was determined at 450 nm using a microplate reader.

### LDH cytotoxicity assay

Lactate dehydrogenase (LDH), an enzyme that released from damaged cells, is a biomarker for the integrity of cell membrane. A LDH cytotoxicity assay kit (Beyotime, China) was used to quantitatively evaluate cellular cytotoxicity according to the manufacturer’s instructions. Briefly, centrifuged supernatant from treated cells was transferred into a new microplate, and then working reaction mixture was added. After incubation at room temperature for 30 min, reactions were stopped and LDH activity was determined by spectrophotometric absorbance at 490 nm. A whole cell lysis mixture treated by LDH release reagent (1:10 dilution, 1 h) was used as a positive control.

### Enzyme-linked immunosorbent assay (ELISA)

Secretion of IL-6, IL-8 and NGF were quantitated in the supernatants by commercial ELISA kits (eBioscience, CA) and read by VERSAmax 96-plate detection system (Molecular Devices, CA) with an absorbed wavelength of 450 nm and a reference wavelength of 570 nm.

### Intracellular reactive oxygen species (ROS) production

After stimulation, HCECs were washed in PBS and treated with 200 μl DMEM/F12 medium and 10 μM H2DCF-DA (Molecular Probes, Invitrogen, 1:1000) for 30 min at 37°. H2DCF-DA probe (2′,7′-dichlorodihydroflulorescein diacetate), a cell-permeable fluorogenic dye, is converted by cellular esterases to non-fluorescent derivative H2DCF that can be oxidized to highly fluorescent 2′,7′- dichlorofluorescein (DCF) in the presence of ROS [28]. The plates were then read at an excitation wavelength of 480 nm and an emission wavelength of 530 nm (Molecular Devices, Sunnyvale, CA).

### Western blot analysis

HCECs were seeded and treated in a 6-well plate, grown to 90% confluence. The main protocol was similar to that described by *Zhang* et al [27]. Briefly, HCECs samples were lysed in RIPA reagent (Beyotime, China) containing a protease inhibitor cocktail (Roche Diagnostics, Germany). Protein concentration in the supernatants was determined by the BCA protein assay (Beyotime, China). Protein extracts were then separated on a 10% SDS polyacrylamide gel by electrophoresis followed by transferring electronically to PVDF membranes. The PVDF membranes were then blocked with 5% nonfat milk dissolved in Tris-buffered saline for 1.5 h at room temperature. After blocking, PVDF membranes were incubated with first antibodies against Phospho-IκBα (1:1000, catalog #2859, CST), Phospho-TLR3 (Tyr759) (1:500, PA5–64722, Invitrogen), Phospho-TrkA (Tyr490) (1:500, catalog #4619, CST), NGF (1:500, ab52918, abcam) at 4° overnight. The membranes were then washed with TBST followed by horseradish peroxidase-conjugated goat anti-rabbit IgG (1:1000, catalog #7074, CST) for 1 h at room temperature. Chemiluminescence solution (ECL, GE Healthcare) was used to reveal the bands, and the bands were quantified by densitometric analysis using M6EV Fluorescent and Imaging System (Peiqing, China). β-actin was used as loading control.

### Immunofluorescence staining

HCECs were cultured on slides placed in 24-well plates (400 μl, 1*10^5^cells/ml). Fixed in 4% paraformaldehyde for 15 min, the cell samples were permeabilized in 0.3% Triton X-100 for 20 min followed by blocking with 3% fetal bovine serum dissolved in phosphate buffer saline at room temperature for 1.5 h. After that, cell samples were incubated with anti-PhophoPlus NF-κB p65/RelA antibody (1:500, catalog #8214, CST) or anti-8-Hydroxy-2′-deoxyguanosine (8-OHdG) antibody (1:200, ab10802, abcam) overnight at 4°, followed by Alexa-Fluor 594-conjugated donkey anti-rabbit secondary antibody. The fluorescent images of the stained cell samples were obtained by a confocal microscope (Leica Microsystems, Germany).

### RNA extraction and quantitative real-time RT-PCR

Total RNA was extracted using the TRIzol reagent (Invitrogen) and reverse transcribed with the PrimeScrip™ RT reagen Kit with gDNA Eraser (Takara RR047A; TaKaRa). Quantitative real-time RT-PCR was performed using primer sequences (Table 1) and QuantiNova SYBR Green kit (Qiagen, Germany). Target gene expression was calculated with the Comparative Ct method, shown as fold change relative to the blank group. GAPDH, a common housekeeping gene, was used as an internal control.

**Table 1:** Primer Sequences for real time RT-PCR

Gene Name	Primer Pair (5′-3′)	NCBI Reference sequence
GAPDH	Fw: 5′-CGACCACTTTGTCAAGCTCA-3′ Rv: 5′-AGGGGAGATTCAGTGTGGTG-3′	NM_002046
MIP1A	Fw: 5′-TGCAACCAGTTCTCTGCATC-3′ Rv: 5′-TTTCTGGACCCACTCCTCAC-3’	NM_001183750.1
MIP1B	Fw: 5’-AAGCTCTGCGTGACTGTCCT-3′ Rv: 5′-GCTTGCTTCTTTTGGTTTGG-3’	NM_001018802.2
IFNB	Fw: 5’-CATTACCTGAAGGCCAAGGA-3′ Rv: 5′-CAGCATCTGCTGGTTGAAGA-3’	NM_002176.3
RANTES	Fw: 5’-AGCAGTCGTCCACAGGTCAA-3′ Rv: 5′-CTTCTCTGGGTTGGCACACA-3’	NM_001278736.

### Statistical analysis

Results are presented as the mean ± SD. SPSS version 18.0 (SPSS, Chicago, IL, USA) was used for statistical analysis. Statistical significance was calculated by one-way ANOVA test or the student’s t-test. A *P* value <0.05 were considered statistically significant.

## Results

### Protective effect of NGF on TLR3-induced cytotoxicity in HCECs

**Fig. 1:**
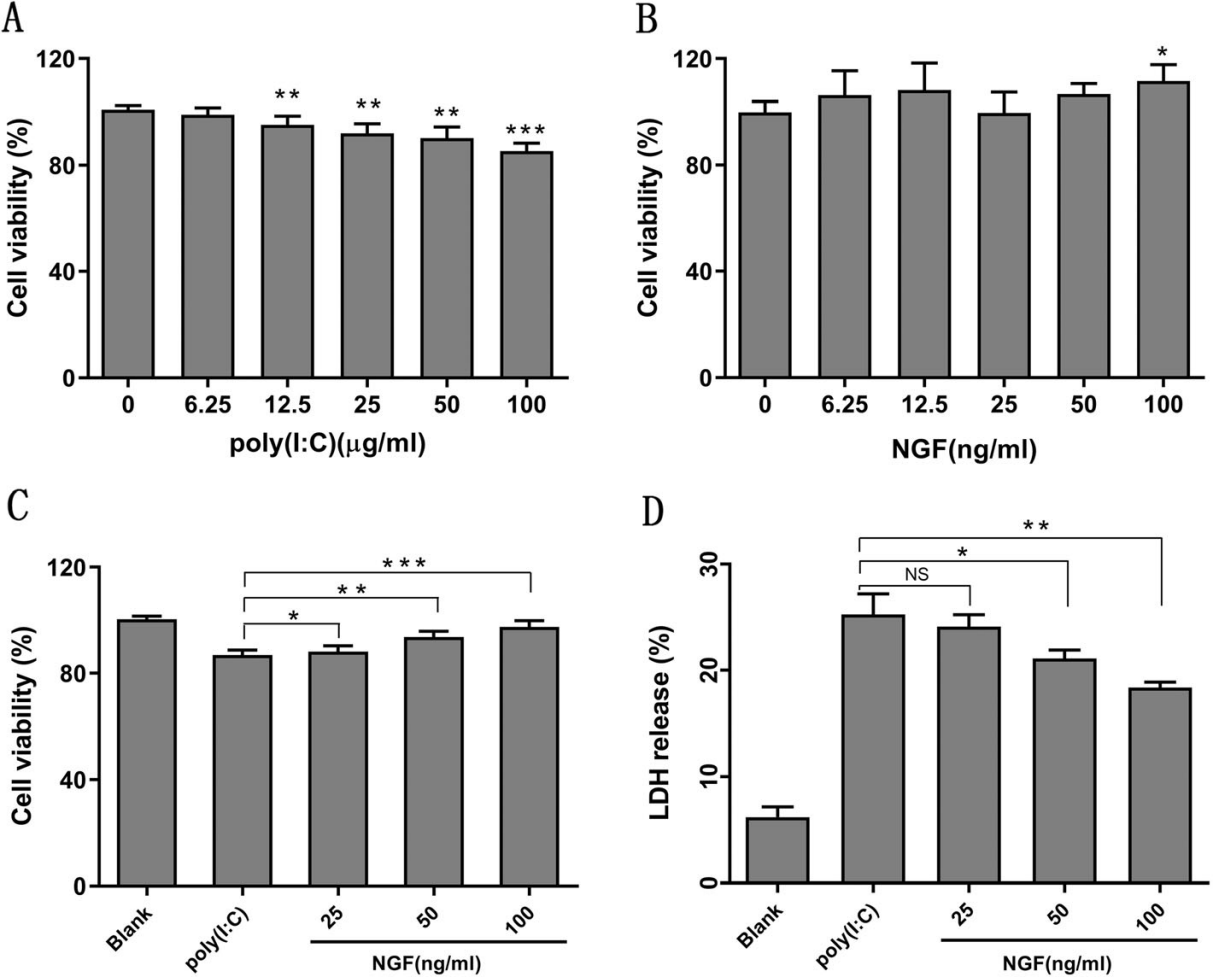
NGF protected cells against TLR3-induced cytotoxicity. **a-b** Cell viability was determined by a CCK-8 assay in HCECs cultured with various concentrations of poly (I:C) or NGF for 24 h. **c** Cell viability was determined by a CCK-8 assay in HCECs co-cultured with NGF (range from 25 ng/ml to 100 ng/ml) and poly (I:C) (25 μg/ml) for 24 h. NGF significantly increased cell viability. **d** Cytotoxicity was determined by a LDH assay when HCECs were co-cultured with NGF (range from 25 ng/ml to 100 ng/ml) and poly (I:C) (25 μg/ml) for 24 h. NGF significantly suppressed LDH release in HCECs. LDH release was presented as percentage of control (whole cell lysis, 100%). Significant differences (^**^p < 0.01, ^***^p < 0.001) existed compared to blank group (without poly (I:C) or NGF), conducted by one-way ANOVA and Dunnett-t test. Significant differences existed between poly (I:C) and poly (I:C) + NGF treatment group, conducted by two-tailed student’s t test. Data represented mean ± SD from six independent representative experiments in each condition

To explore the protective role of NGF on TLR3-induced cytoxicity, HCECs were stimulated with various concentrations of poly (I:C) in the absence or presence of NGF. Cell viability was determined by a CCK-8 assay, as well as a LDH release assay. It was shown that poly (I:C) stimulation significantly reduced cell viability in a dose-dependent manner (Fig. 1a), while NGF supplement showed protective effect especially at maximal concentration (100 ng/ml) (*P* < 0.05, Fig. 1b). We next tested the effect of NGF toward TLR3-induced cellular cytoxicity while HCECs were simultaneously cultured with poly (I:C) (25 μg/ml) and NGF (ranging from 25 ng/ml to 100 ng/ml) for 24 h. It was shown that NGF treatment could significantly increase cell viability (Fig. 1c) and decrease LDH release (Fig. 1d) in a dose-dependent manner, indicating the protective effect of NGF on HCECs against TLR3-induced cytotoxicity.

### NGF directly inhibited downstream NF-κB activation in HCECs

**Fig. 2:**
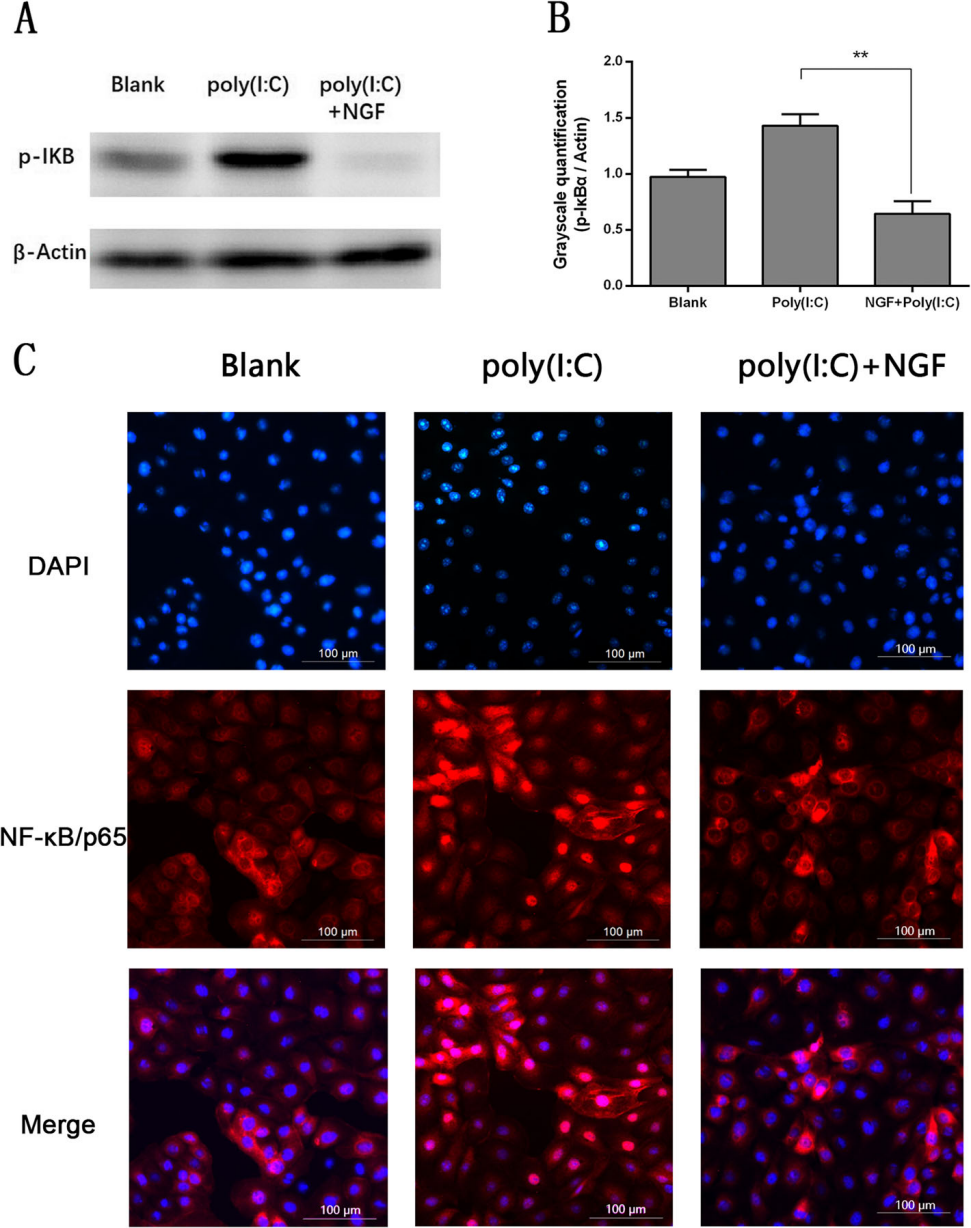
NGF directly suppressed NF-κB activation triggered by poly (I:C) stimulation in HCECs. **a** The phosphorylation level of IκBα was downregulated after coincubation with NGF (50 ng/ml) in response to poly (I:C) (25 μg/ml) stimulation for 10 h. **b** Histogram represented quantified data depicted in A. **c** The nuclear translocation of NF-κB/p65 in response to poly (I:C) (25 μg/ml) stimulation was inhibited by NGF (50 ng/ml) treatment for 10 h. Representative data of three independent experiments and quantitative densitometry results were shown

NF-κB signaling is a critical downstream target of TLRs in inflammatory pathways through regulating the expression of a series of inflammation related genes. Once IκBα is phosphorylated, it releases and activates NF-κB/p65, which transfers from the cytosol into the nucleus to regulate gene transcription [29]. Therefore, we examined whether NGF could prevent IκBα phosphorylation and subsequent nuclear translocation of NF-κB/p65. As shown in Fig. 2, poly (I:C) stimulation could significantly enhance IκBα phosphorylation and boost the nuclear translocation of NF-κB/p65 in HCECs, while such effect was substantially inhibited by cotreatment with NGF.

### NGF suppressed the production of downstream pro-inflammatory cytokines in HCECs induced by TLR3 activation

**Fig. 3:**
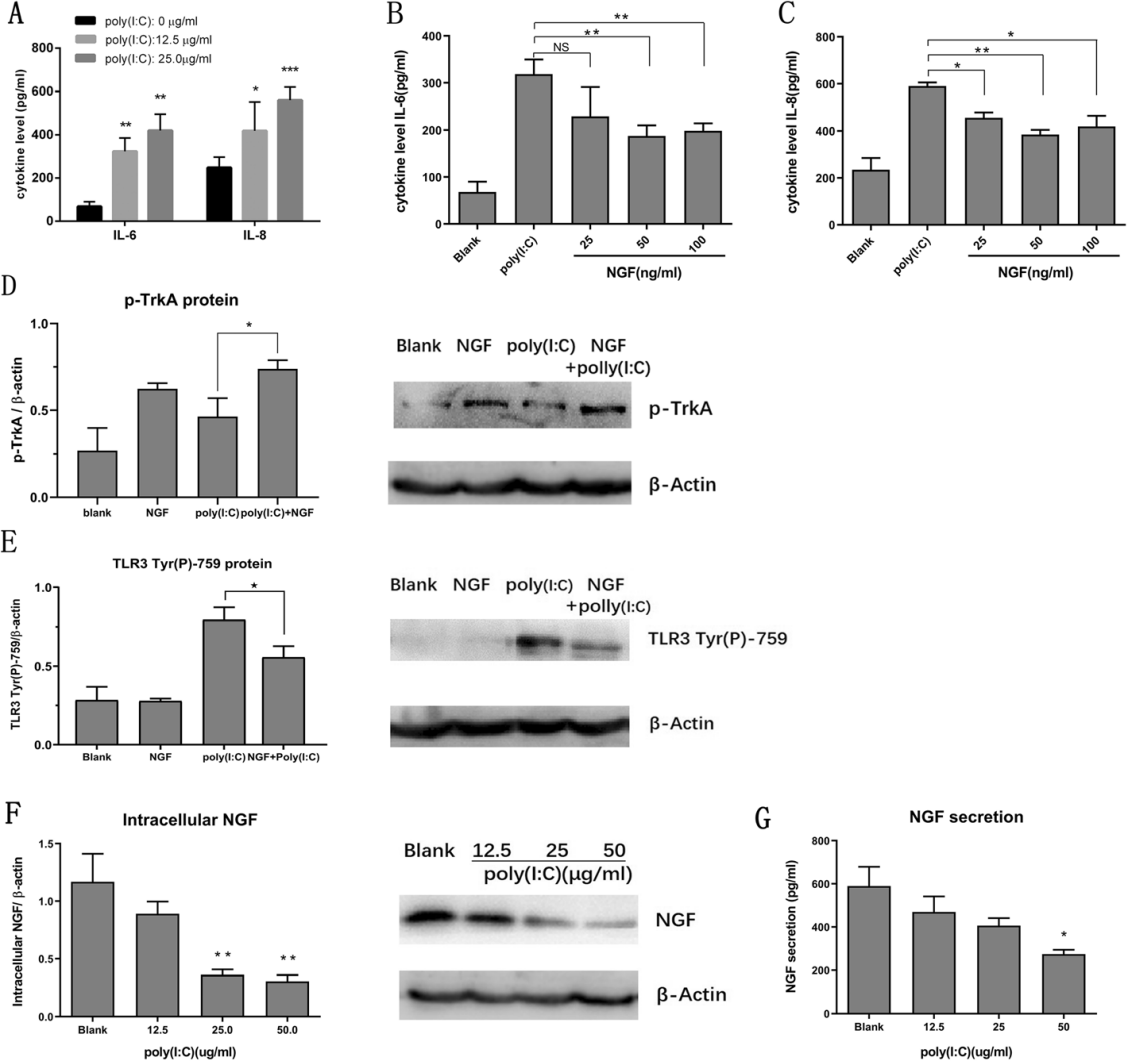
NGF suppressed the production of proinflammatory cytokines in HCECs induced by TLR3 activation. **a** Increased secretion of IL-8 and IL-6 in HCECs stimulated with poly (I:C) for 24 h in a dose-dependent manner. **b-c** Various concentrations of NGF could suppress the secretion of IL-6 and IL-8 in HCECs treated with 25 μg/ml poly (I:C) for 24 h. **d** TrkA phosphorylation was strongly increased after coincubation with NGF (50 ng/ml) in response to poly (I:C) (25 μg/ml) stimulation for 24 h. **e** TLR3 phosphorylation was decreased after coincubation with NGF (50 ng/ml) in response to poly (I:C) (25 μg/ml) stimulation for 1 h. **f** Reciprocally, TLR3 activation decreased intracellular NGF protein levels in HCECs treated with poly (I:C) for 24 h. **g** Stimulation with poly (I:C) for 24 h decreased NGF secretion by HCECs in a dose-dependent manner. Data represented mean ± SD from three independent representative experiments in each condition

Next, we measured the secretion of downstream pro-inflammatory cytokines following TLR3 activation. In agreement with previous study [11], exposure to various concentrations of poly (I:C) remarkably increased the secretion of IL-8 and IL-6, in a dose-dependent manner (Fig. 3a). However the elevation of pro-inflammatory cytokines were significantly suppressed by NGF treatment with concentrations ranged from 25 ng/ml to 100 ng/ml (Fig. 3b, c). Notably, treatment with 100 ng/ml of NGF was found to slightly induce cell proliferation (Fig. 1b), and thereby the suppressive effect of NGF on this concentration was relatively weaker probably due to the increasing cell numbers. Therefore, HCECs were treated with NGF (50 ng/ml) and poly (I:C) (25 μg/ml) to further elucidate the role of NGF on TLR3-induced cellular inflammation. The phosphorylation of NGF receptor, TrkA (Tyr-490) was significantly increased, while TLR3 (Tyr-759) phosphorylation was substantially inhibited in response to NGF treatment in TLR3 activated HCECs (Fig. 3d, e). Reciprocally, TLR3 activation was found to hamper NGF expression in a dose-dependent manner. Both intracellular NGF and secreted NGF were significantly decreased upon exposure to various concentrations of poly (I:C), suggesting downregulation of NGF expression in response to TLR3-induced innate immunity (Fig. 3f, g).

**Fig. 4:**
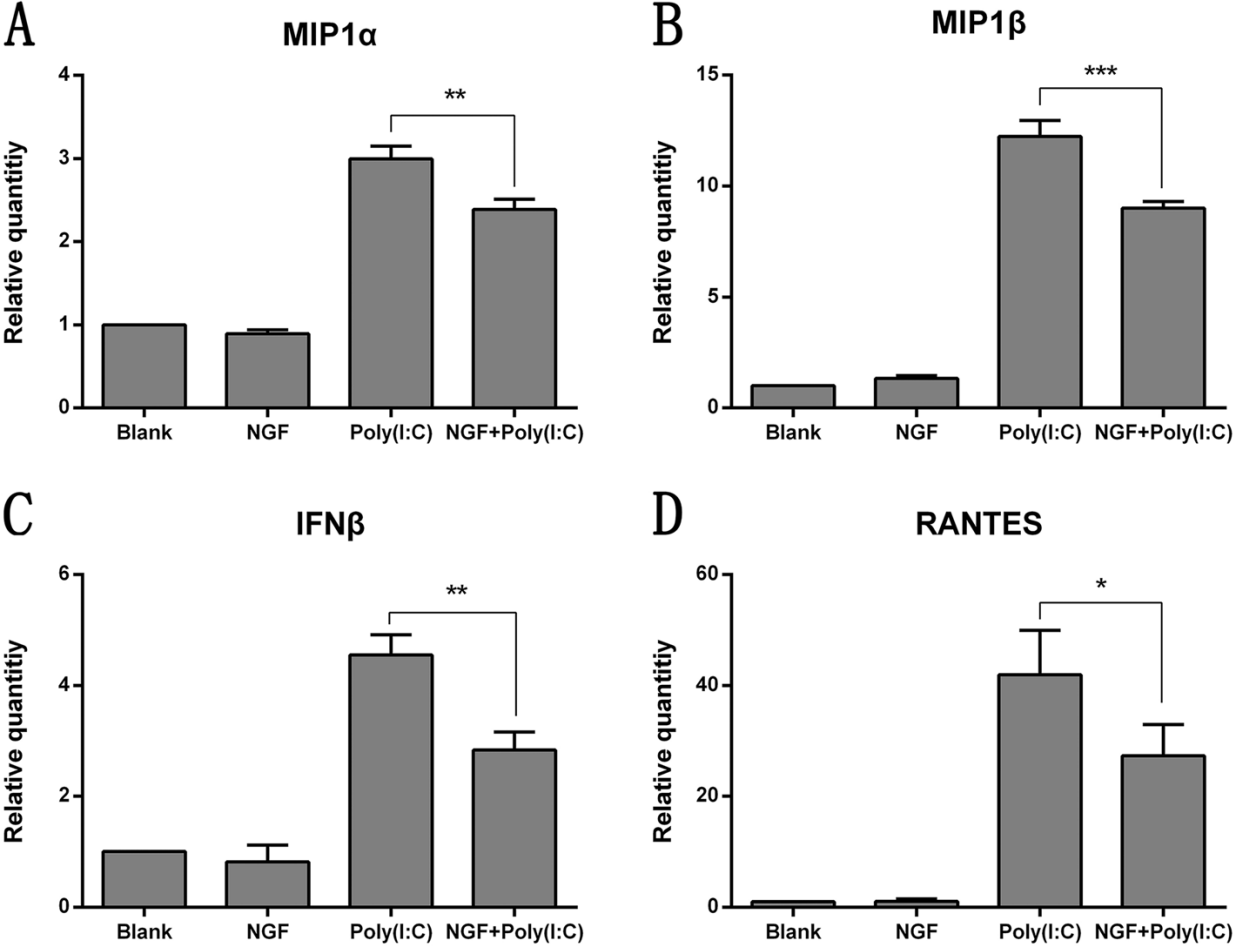
NGF suppressed the expression of pro-inflammatory mediators in HCECs exposed to poly (I:C) stimulation. **a-d** Real time RT-PCR analysis of mRNA levels in HCECs after exposure to poly (I:C) (25 μg/ml) in absence or presence of NGF treatment (50 ng/ml) for 18 h. NGF (50 ng/ml) suppressed the gene expression of pro-inflammatory mediators including *IFNβ, MIP1α, MIP1β* and *RANTES* in HCECs treated with 25 μg/ml poly (I:C). Data represented mean ± SD from three independent representative experiments in each condition

It was also observed that the gene expression levels of some pro-inflammatory cytokines and chemokines including *IFNβ, MIP1α, MIP1β* and *RANTES* were also significantly inhibited by NGF pretreatment (Fig. 4a-d).

### NGF attenuated TLR3-induced ROS overproduction in HCECs

**Fig. 5:**
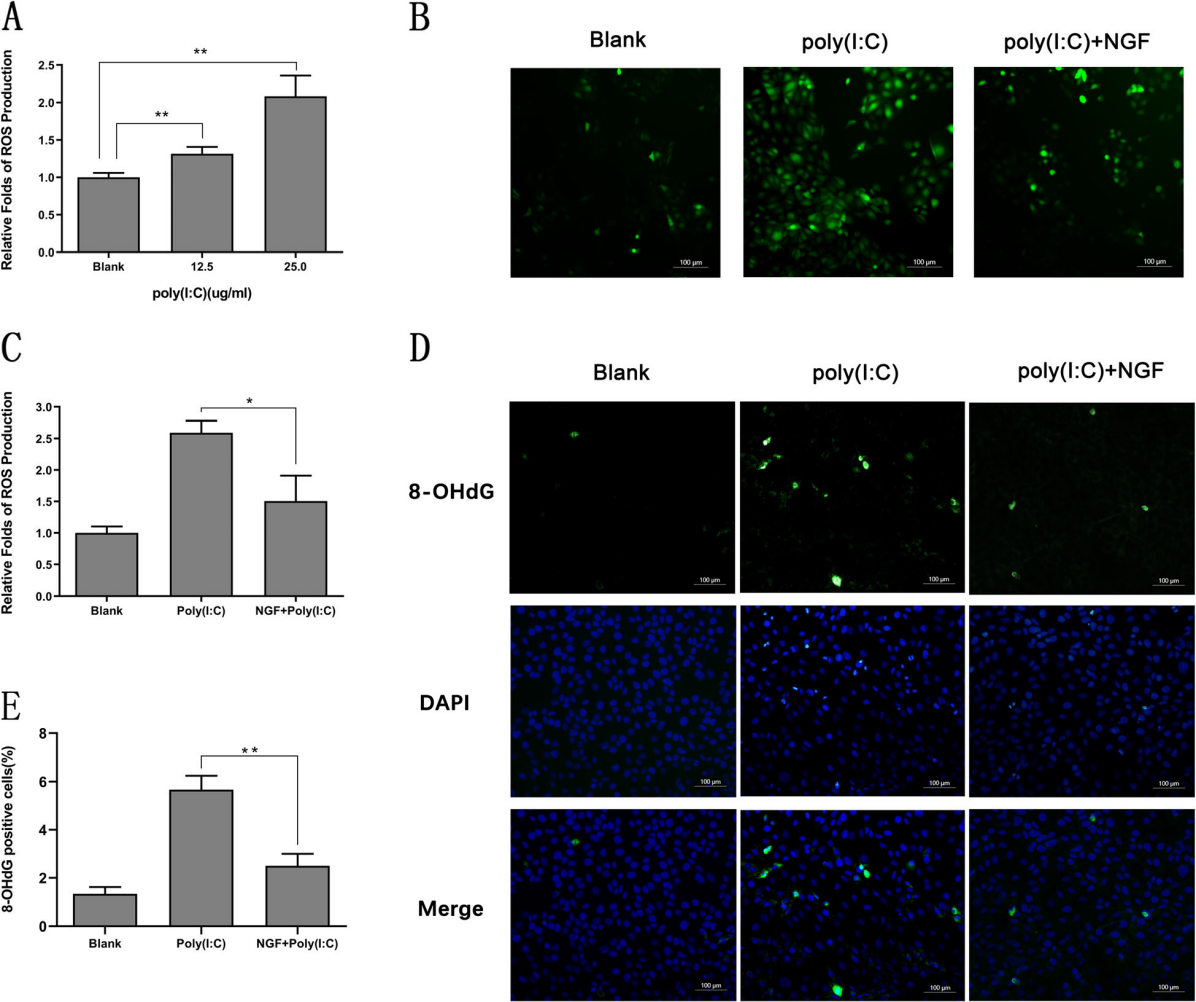
NGF attenuated TLR3-induced ROS overproduction in HCECs. **a** Increased generation of ROS in response to various concentrations of poly (I:C), detected by H2DCFDA probe. The fluorescence intensity was quantitatively measured as mean ± SD from three independent experiments in each condition. **b-c** ROS generation in cells treated with/without NGF (50 ng/ml) in response to poly (I:C) (25 μg/ml) stimulation for 10 h. C represented quantified data depicted in B. DCF fluorescence intensity was shown as fold change comparing to that of untreated cells. (**d-e**) NGF (50 ng/ml) treatment decreased the number of 8-OHdG positive cells in response to poly (I:C) (25 μg/ml) stimulation, detected by immunofluorescent staining . Images were representative for each of three experiments, taken at 200 × magnification

It was observed that TLR3-triggered ROS generation was required for NF-κB activation in monocytes. Moderate ROS generation could lead to a physical association between components of the NADPH oxidase enzyme complex, which were required for the phosphorylation and nuclear translocation [14]. In the next setting, we investigated whether NGF presented potential roles on modulating this upstream signal of initiating NF-κB activation. The result confirmed that poly (I:C) stimulation could promote ROS generation by HCECs, in a dose-dependent manner, especially at the concentration of 25 μg/ml (Fig. 5a, *P* <0.01). However, co-incubated with NGF remarkably scavenged these overexpressed ROS by approximately 50% (Fig. 5b, c, *P* <0.05). Moreover, ROS can react with nucleic acids inducing single- and double-stranded DNA breaks, namely, ROS-induced 8-hydroxylation of the guanine base (8-OHdG) in nuclear DNA [30]. We next examined the level of 8-OHdG to further investigate whether the high level of TLR3-induced ROS generation led to irreversible damage to nuclear DNA. An obvious increase of 8-OHdG positive cells was observed in poly (I:C)-treated group, whilst such elevation was significantly inhibited by NGF treatment (Fig. 5d, e).

## Discussion

In the current study, we triggered TLR3 activation with poly (I:C) in HCECs, mimicking one of the key steps of HSK induced corneal injury in vitro*.* The results demonstrated that NGF showed protective effect against TLR3-induced inflammation. NGF significantly inhibited the generation of proinflammatory cytokines in HCECs that were triggered by TLR3 activation, probably via directly suppressing NF-κB activation. NGF also impeded the upstream signal to initiate NF-κB activation by scavenging ROS. Collectively, it was demonstrated firstly that NGF could ameliorate HSK associated corneal injury in vitro, highlighting NGF as a potential therapeutic agent for HSK.

Growing evidence showed that NGF played a critical role in the inflammatory response in a variety of cells. Our results showed that intracellular NGF production and secreted NGF were both decreased in response to TLR3 activation, which exacerbated abnormal secretion of inflammatory cytokines IL-6 and IL-8. In contrast, addition of NGF could attenuate it. The effect of NGF in our situation was in accordance with previous observations found in LPS-activated monocytes [31] and high glucose-treated HCECs [24] in the presence of NGF. Indeed, NGF signaling existed at the top of a hierarchical network, controlling cell cycle, Toll-like receptor, senescence regulatory pathways and VEGF signaling [32]. Besides its effect on regulating innate immunity via TLRs, NGF could promote cell cycle progression of HCECs by regulating D-type cyclins via PI3K/Akt and MAPK/Erk activation [33, 34]. NGF was also shown as a key promoter of limbal stem cells proliferation, colony-forming efficiency, and a maintainer of the LSC phenotype [32]. Moreover, the effects of NGF on promoting corneal epithelial and nerve regeneration, as well as corneal healing have been reported both in vitro and in vivo [19, 35, 36]. All these evidences underlined an important role of NGF in corneal physiopathology, and also suggested that NGF might exert therapeutic action in a wide spectrum of corneal diseases.

It was shown that HSV-1 infection quickly induced intracellular ROS that were necessary for proper activation of innate antiviral immune responses [37]. Initial generation of ROS post virus infection acts as a microbicidal compound to regulate several processes, such as expression of innate-response-related genes, NF-κB activation, inflammasome activation, autophagy and programmed necrosis [38–40]. However, its overproduction may exacerbate downstream inflammatory responses as well as oxidative injury depending on the absolute intracellular levels of ROS and other reactive species [16, 41]. Our results showed high levels of ROS generation and 8-OHdG, a marker of ROS-induced DNA breaks, in response to TLR3 activation. Previously, it has already been demonstrated that NGF could inhibit oxidative injury that was induced by hyperosmotic stress [42, 43] or high glucose levels [24] indicating the remarkable capacity of NGF on scavenging ROS generated during the pathogenesis of dry eye or diabetic cornea. Consistently, the current study also demonstrated the suppressive role of NGF on HSK related ROS generation, implicating that the underlying mechanisms of NGF on treating these three common corneal diseases might be partially overlapped.

Previously, *Li* et al. proved that signaling pathways including NF-κB, JNK and p38 were all activated by HSV-1 infection in HCECs. Interestingly, these signaling pathways underwent two phases of activation in response to infection [44]. It was shown that HSV-1 could induce TLR7 expression at later phase after virus infection, and the induced TLR7 expression was coincident with the declined expression of TLR3, implicating that TLR3 and TLR7 might act sequentially to response to viral infection and replication in HCECs [3]. In this study, the in vitro results using TLR3 agonist-induced model in HCECs may not fully reflect the real situation in HSK. We only focused on the initial step of inflammation responses that triggered by TLR3 activation. The interplay between NGF and other HSK-related TLRs, as well as the underlying mechanisms requires in-depth investigations before its further extension to in vivo clinical conditions.

## Conclusion

In summary, we demonstrated here for the first time that NGF inhibited TLR3-induced inflammatory cascades in HCECs. The present study indicated that NGF could be a promising therapeutic agent in clinical treatment of HSK.

## Data Availability

All data generated or analyzed during this study are included in this published article.

## Abbreviations

8-OHdG: anti-8-Hydroxy-2′-deoxyguanosine
Fw: Primer forward
GAPDH: Glyceraldehyde-3-Phosphate Dehydrogenase
HCECs: Human corneal epithelial cells
HSK: Herpes simplex keratitis
HSV-1: Herpes simplex virus 1
IFNβ: Interferon Beta
MIP1A: C-C motif chemokine ligand 3
MIP1B: C-C Motif Chemokine Ligand 4
NCBI: National Center for Biotechnology Information
NGF: Nerve growth factor
RANTES: C-C Motif Chemokine Ligand 5
ROS: Reactive oxygen species
Rv: Reverse forward
TLR3: Toll like receptor 3
